# Resection of Calcified and Giant Thoracic Disc Herniation Through Bilateral Postero-Lateral Approach and 360° Cord Release: A Technical Note

**DOI:** 10.3390/jcm13226807

**Published:** 2024-11-12

**Authors:** Ismail Zaed, Benjamin Pommier, Gabriele Capo, Cédric Y. Barrey

**Affiliations:** 1 Department of Spine Surgery, Hôpital Pierre Wertheimer, Hospices Civils de Lyon, and Claude Bernard University of Lyon 1, 69367 Lyon, France; 2 Department of Neurosurgery, Neurocenter of Southern Switzerland, Ente Ospedaliero Cantonale, 6900 Lugano, Switzerland; 3 Laboratory of Biomechanics, ENSAM, Arts et Metiers ParisTech, 75013 Paris, France

**Keywords:** thoracic disc herniation, herniated disc, surgical approach, thoracic hernia, technical note

## Abstract

**Background/Objectives:** Surgical treatment of thoracic disc herniation (TDH) is risky and technically demanding due to its proximity to the spinal cord and the high possibility of the TDH being calcified (up to 40%), making the resection even more complex. Calcified TDH may be resected from an anterior via thoracotomy/thoracoscopy, lateral extra-cavitary, or a postero-lateral approach. Here, we present our experience in managing such pathology with an original technique **Methods:** This original technique, used successfully in more than 40 patients, is introduced, with a precise description of the surgical anatomy and the surgical steps to take. Indications for surgical management and neurological outcomes are also analyzed. This surgical approach consisted of transverso-pediculectomy, most often bilaterally, partial vertebral body drilling, 360° release of the cord, and short fixation. **Results**: A total of 44 patients were collected, with a mean age of 52.4 ± 11.7 years. Seven patients (15.9%) had complete calcifications, and thirty-one had partial calcifications (70.5%), while the remaining six did not have signs of calcifications. There were only 4 intraoperative complications (2 dural tears and 2 loss of evoked potentials). The TDH could be resected in total for 39 patients (88.6%) and partially, according to the “floating” technique, in 5 patients (11.4%). In the postoperative follow-up, all of the patients except two (presenting with sensory aggravation) reported an improvement in neurological conditions leading to an overall risk of neurological aggravation of 4.5%. **Conclusions:** The bilateral postero-lateral approach provides a large decompression of the cord (360°) and gives safe access to the TDH, even calcified, permitting high rates of total resection. It also prevents any prejudicial pressure on the spinal cord, reducing the risk of severe postoperative deficits and permitting optimal instrumentation (pedicle screw-based) of the spinal segment. The surgical sequence to resect the bony structures around the spinal cord is of great importance.

## 1. Introduction

Symptomatic disc herniation in the thoracic spine (TDH) is quite a rare pathology since it is reported to affect from 1 in 1000 to 1 in 1,000,000 people in the general population, accounting for 0.1 to 3% of all spinal disc herniation [1]. On the contrary, the presence of asymptomatic TDH on imaging examinations is much more frequent and has been estimated from 11% to 37%. TDH is most common in adults from 30 to 50 years of age, with equal distribution between the two genders [2]. Concerning the localization, in 75% of cases, the TDH is located below the T7–T8 disc, with only 4% of TDHs located above T3–T4 and T11–T12 being the most frequently affected level. The vulnerability of the T11-T12 disc is supposed to be related to its greater mobility and posterior longitudinal ligament weakness at this level [2,3]. Cases of TDH complicating proximal junctional syndrome after thoracolumbar fusion have also been reported [1].

In up to 40% of cases [1], TDHs are calcified resulting in adherences between TDH and the dura and a higher risk of both dural tears and neurological deterioration after surgery.

Concerning the treatment, despite having a well-known natural history and clinical evolution, its management remains still a topic of discussion [2]. Surgery for TDH has a poor reputation because of its technical difficulties and the risk of potentially serious and hard-to-treat complications (including neurological deterioration and dural tear). The TDH may be resected via an antero-lateral or postero-lateral approach. Up to now, there has been no clear evidence of which approach is “superior” to the others, each being associated with advantages and limitations [1,2,4,5].

In this study, we presented an original surgical technique, used routinely in our department, to treat symptomatic, giant, and/or calcified TDH through a postero-lateral approach with transverso-pediculectomy and partial vertebral body drilling, performed most often bilaterally, and associated with short fixation. We also discussed the results of a clinical series of patients treated consecutively with this technique.

## 2. Material and Methods

### 2.1. Patient Selection

This study is based on a consecutive clinical series of patients treated. The epidemiological unit of the hospital has been contacted to retrieve all the identification information of all the patients treated in the last 10 years (from January 2010 to December 2021) for a thoracic disc herniation, using specific administrative codes for TDH.

A total of 44 patients with a diagnosis of giant and/or calcified thoracic disc herniation were retrieved. All the patients included underwent a microsurgical resection of TDH by the senior neurosurgeon of the department.

All patients that did not respect the inclusion and exclusion criteria were not considered in the analysis.

The study was conducted according to the Declaration of Helsinki. Informed consent was obtained from all participants.

### 2.2. Inclusion Criteria and Exclusion Criteria

Patients aged ≥ 18 years with TDHs surgically treated at our institution between January 2010 and December 2021 were included. Only giant (defined as an occupation ratio ≥ 40%) and/or calcified (partial/total) TDHs were included. All patients needed to have both preoperative MRI and CT scan and a minimum of 6 months of postoperative follow-up. Patients without a complete clinical assessment or missing imaging data were excluded from the study.

The surgical indication is decided upon when the patient has functional symptoms that do not respond to medical treatment and/or when neurological symptoms appear or worsen. The presence of myelopathy signs on MRI, even in the absence of clinical neurological signs, can be a surgical indication in our practice, before these signs appear and become irreversible.

### 2.3. Data Collection

Data related to the selected population were extracted by 2 independent investigators (IZ and BP). Patients’ data were reconstructed based on clinical assessments (in the preoperative and postoperative period and at the last follow-up), radiographic images (preoperative, intraoperative, postoperative, and at last follow-up), and operative notes.

All the operative notes were written by the senior author. All radiographic images were revised by a senior neuro-radiologist. No outside medical records were used.

### 2.4. Surgical Technique

**Figure 1:**
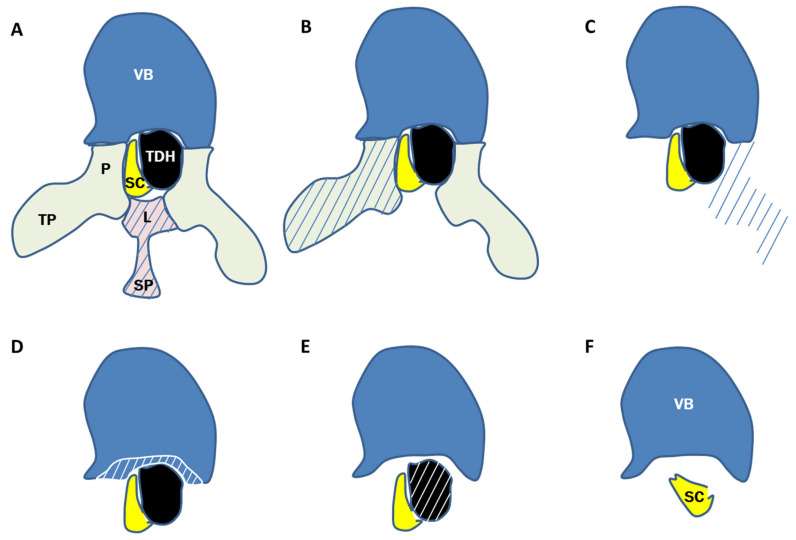
Surgical steps of the proposed approach for thoracic herniation removal: (**A**) Resection of the lamina and spinous process (drilling); (**B**) resection of the transverse process and pedicle at the cord side (drilling); (**C**) resection of the transverse process and pedicle at the TDH side (drilling); (**D**) decompression in front of SC and TDH (drilling); (**E**) progressive removal of the TDH with gentle separation between SC and TDH (rongeur/drill); (**F**) 360° decompression of the cord with complete resection of the TDH is achieved.

The following steps should be undertaken to remove the TDH. The most important steps below described are illustrated in the figure (Figure 1).

The patient should be positioned in a classic prone position under general anesthesia and after placement of electrodes for multimodal intraoperative neurological monitoring (IONM) including MEPs, SSEPs, and D-wave.

A central skin incision is made at the level of the herniation. The exact level could be found with the help of fluoroscopy.

An incision of the thoracic aponeurosis is made to identify the inter-laminar space at the level of the herniation and expose the posterior bony elements at the index level and adjacent levels (*n* + 1 and *n* − 1).

Once the correct level is confirmed with fluoroscopy, a short fixation should be performed with pedicle screws between *n* + 1 and *n* − 1 vertebras. The fixation is extended to more levels in case of significant kyphosis and/or Scheuermann disease. Also, if the compression is very severe with the spinal cord seeming highly vulnerable, we recommend placing spinal implants after TDH resection to reduce the risk of spinal cord injury during pedicle screw placement.

The next step is the opening of the spinal canal which is started far away above and below the place of maximal compression by the TDH. The spinous process removal together with 1/3 laminectomy of the vertebra above and below the herniation avoid the zone of the TDH at the early stage. It is important to partially remove the lamina, on both sides for most cases, in order to adequately enlarge the spinal canal posteriorly and expose the spinal cord. The partial laminectomy may be realized using a soft or diamond drill under water irrigation to avoid the need to insert any instrument inside the canal and minimize the risk of cord injury (Figure 1A).

Removal of the transverse process of the first side (cord side) is then achieved with progressive drilling of the ipsilateral pedicle. The objective is to remove all the bony structures laterally to the cord coming obliquely with no or minimal manipulation of the dural sac. Inferior and superior facets are resected using rongeur and/or osteotome (Figure 1B). The pedicle is finally resected in total with the drilling to be continued through the posterior part of the vertebral body in the direction of the disc.

Similarly, on the opposite side (TDH side), we proceed with the removal of the transverse process and drilling of the pedicle (Figure 1C).

All the bony structures are thus resected around the cord to obtain a 360° release of the spinal cord, thus limiting the risk of compressing the cord against any bony structures (against the pedicles in particular) and allowing the gentle mobilization of the spinal cord if necessary.

The drilling is then conducted through the pedicle traversing the posterior part of the vertebral body to the disc space in front of the cord and the TDH with the ultimate objective to completely separate the TDH from its ligament attachments (Figure 1D).

At this step, progressive removal of the disc and part of the hernia can be achieved safely with the spinal cord largely released and no more bone around. Gentle mobilization of TDH is now possible, pushing it anteriorly inside the bony cavity created and detaching the TDH from the PLL cranially and caudally.

According to the topography, the size, and the adherence, it is good to work each side of the cord in alternance. Discectomy and removal of the herniation are then finalized (Figure 1E,F).

In case of major adherence(s) between TDH and the dura, it may be preferable to leave a small part of the DH against the dura to avoid the risk of dural tear and/or spinal cord injury (according to the concept of the “floating technique”) [2,3].

**Figure 2:**
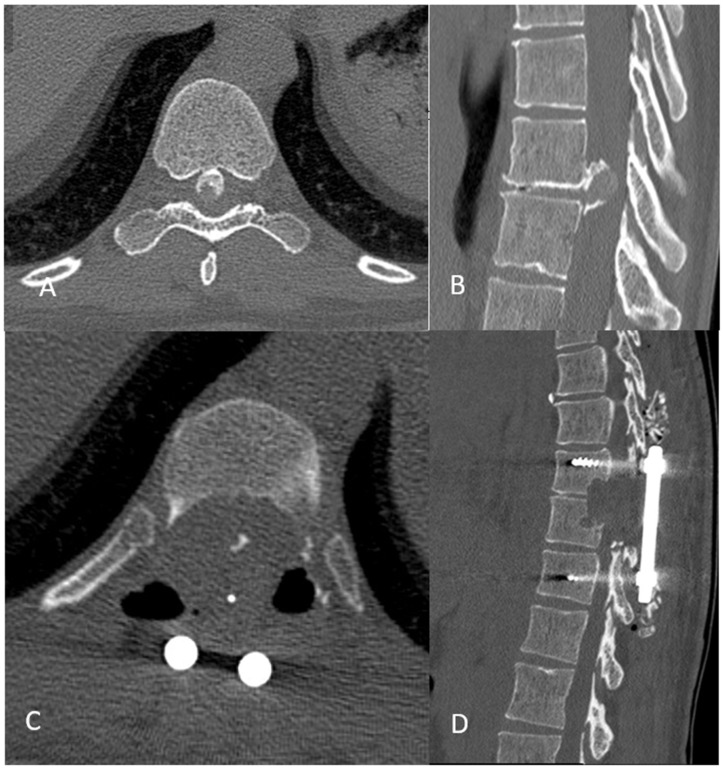
Axial (**A**,**C**) and sagittal view (**B**,**D**) of a pre- and postoperative scanner.

Once TDH is resected and the spinal cord adequately decompressed, the definitive rods are positioned with the completion of the arthrodesis (Figure 2).

In our experience, SSEPs, MEPs, and D-wave are useful to guide the surgeon during the different steps of the surgery and confirm the tolerance of the spinal cord during the procedure.

### 2.5. Statistical Analysis

Data were analyzed using a statistical package for social sciences (SPSS) version 20.0 (IBM, Armonk, NY, USA). In particular, categorical variables were expressed as number (*n*) and percentage, and quantitative variables were expressed as means ± standard deviation. Continuous variables were compared using an independent samples *t*-test.

An alpha value of *p* < 0.05 was accepted as statistically significant.

## 3. Results

A total of 44 patients met the inclusion criteria for the study. All demographic and surgery data are summarized in Table 1. Out of the total series, 25 patients (56.8%) were female and the remaining 19 (43.2%) were male. The mean age of the patients at the moment of the surgery was 52.4 ± 11.7 years. Among the patients, 12 (27.3%) were obese with a BMI ≥ 30.

Regarding the level of disc herniation, a minority of them were located in the upper thoracic spine (4 patients, 9.1%), whereas the majority were mostly equally divided between the middle thoracic spine (19, 43.2%) and the lower thoracic spine (21, 47.7%). All data are summarized in Table 2.

Concerning the clinical signs at the presentation, 6 patients (13.6%) had no significant neurological deficit but radicular pain, 7 patients (15.9%) had an important sensitive deficit, and the remaining 31 (70.5%) also had a motor deficit with claudication. The severity of the signs was evaluated according to the ASIA impairment scale, summarized in Table 3. The mean duration of the symptomatology prior to the diagnosis was 19 months (6–48 months).

From a radiological point of view, all of them were giant disc herniation. Out of the total series, 7 patients (15.9%) had complete calcifications, 31 (70.5%) had partial calcification, and the remaining 6 patients (13.6%) did not show any sign of calcification on the preoperative CT scan.

All patients were operated on using multimodal IONM including MEPs, SSEPs, and D-wave. No intraoperative major complications were reported, see Table 1. We had two dural tears and two sensory neurological deteriorations but no paraplegia. No hematoma or CSF leak was seen. No reoperations were necessary for complications and/or mechanical failure.

In the multivariate analysis, no correlation was found between the level operated and the postoperative complication, whereas a statistical correlation was highlighted between the grade of calcification and the risk of dural tears.

The mean length of stay was 6.6 days (3–50 days). The mean follow-up was 11.7 months (range 6–25 months). At the last follow-up, all patients except two reported a reduction or a complete resolution of preoperative neurological symptoms.

## 4. Discussion

Because of the underlying anatomy, neurosurgical management of the TDH is a technical challenge, despite recent technological advances. Key drivers of preoperative decision-making include the anatomic location and consistency of the herniated disc, the patient’s functional status, and the surgeon’s experience [6].

In the present study, we described the surgical technique used in a tertiary center in the management of TDH. The current study analyzed 44 patients. Overall, the surgical approach described seems to present a lower rate of postoperative complications compared to the most traditional anterior and posterior approaches [1,4,7,8,9,10]. In our experience, we had two complications: a dural tear during the TDH’s removal that has been sutured without any postoperative issue and a transient neurological deficit in the immediate postoperative time that recovered completely in the next few days.

The risk of a dural breach is reported to be around 15% in anterior approaches resulting sometimes in catastrophic late complications [11]; this, however, should be greatly minimized in the posterior approaches. More importantly, the management of dural tears after the posterior approach is much simpler. A calcified shell should be left on the dura if it is strongly attached to the hernia. At the end of the procedure, any breach should be sealed by direct suture, or preventative application of biological glue to the dura, filling dead spaces against the dura with muscle or aponeurosis tissue and closing the posterior parietal pleura if possible.

There is no consensus as to the need for IONM during thoracic discectomy [12]. Two groups reported a reduction in the risk of permanent neurological damage when using somatosensory evoked potentials (SSEPs) and recommended their use [10,13]. In a 2007 expert opinion, the following indication was proposed for intraoperative motor evoked potentials (MEPs) in spine surgery: extensive anterior and/or posterior decompression in spinal stenosis in the cervical, thoracic, and lumbar spine causing myelopathy or cauda equina syndrome. However, this indication is controversial [14]. The intraoperative monitoring of the dorsal spinal cord during a TDH procedure is desirable, especially when the herniation is large and calcified. If intraoperative evoked potentials are used, testing these potentials preoperatively is useful as a reference and to identify early spinal cord pathology.

We believe that multimodal IONM of the dorsal spinal cord during a TDH procedure, combining both SSEPs and MEPs, is desirable, especially when the herniation is large and calcified. If intraoperative evoked potentials are used, testing these potentials preoperatively is useful as a reference and to identify early spinal cord pathology [15,16,17].

Permanent postoperative deterioration of the neurological condition has been reported in all studies, no matter which approach is used, at a frequency of 2% to 5%^2^. The most at-risk patients are those with a giant calcified TDH who have neurological signs preoperatively. The clear pathogenesis of these deficits is unknown, although several hypotheses have been suggested: medullary inhibition, spinal shock, medullary contusion, or vascular problem. Based on our experience of more than 40 cases, we can make several recommendations to prevent these neurological deficits:

(1) Preoperative screening with a good MRI and CT scan is crucial to determine the degree of calcification and the precise location of the cord inside the canal and its relation with the TDH.
(2) In the case of giant calcified TDH, a total release of the cord, 360°, is essential to remove all bony surrounding structures and reduce the risk of injury by pushing the cord against the contralateral pedicle.
(3) We recommend releasing first the cord side. If not, there is the risk of increasing the compression of the cord between the TDH and the contralateral pedicle.
(4) It is also advised to drill progressively on each side of the cord in alternative (painstaking work).
(5) In case of major adherence between TDH and the dura, it is preferable to leave a small part of the DH against the dura, “floating technique”, to avoid the risk of dural tear and/or spinal cord injury.
(6) Finally, the French Anaesthesia and Intensive Care Society (SFAR) recommendations for medullary protection must be followed, particularly the need to maintain an average blood pressure of more than 80 mmHg during and after the surgical procedure. It has been also proposed to inject high doses of corticosteroids at the start of the procedure and maintain an average blood pressure of 80 mmHg [18,19,20].

### 4.1. Advantages over Anterior Approaches

Anterior techniques have been popular for the management of TDHs since the studies published by Rosenthal et al. in the 1990s [21], although there is no clear advantage of one technique over the others. A recent investigation of the American College of Surgeons—the National Surgical Quality Improvement Program (ACS-NSQIP)—found no statistical difference concerning discharge disposition. In light of their findings, they suggested that surgeons should weigh the risks and benefits of each surgical technique during the decision-making process [4,22].

Posterior techniques remain relevant and are associated with significant advantages:

(1) Easier control of epidural bleeding;
(2) Lower risk of dural tear and easier management if it happens;
(3) 360° decompression of the cord;
(4) Optimal stabilization of the thoracic segment based on pedicle-screw constructs;
(5) A posterior approach is more familiar to most spine surgeons.

### 4.2. Study Limitations

Despite the authors’ best efforts, the present study has some limitations, mainly related to the nature of the analysis, since it is a retrospective study. The overall quality of the data will be improved in the case of a prospective study. Another limitation is related to the fact that the results presented are operator-dependent since all cases presented were performed by one surgeon. Further comparative studies should validate the technique here presented. Another possible limitation is the length of the follow-up. The study will benefit from a longer follow-up.

## 5. Conclusions

TDH is a rare pathology with several different clinical manifestations, with neurological deficits being the most feared. TDH is difficult to treat and surgical management can provide a great number of complications.

Our posterior approach for the treatment of TDH with 360° cord release seems to be safe and effective, with fewer complications compared to the general rate. Future studies should verify our conclusions in other clinical series.

## Figures and Tables

**Table 1:** Demographic and surgery data of patients included in the study.

Patients Characteristics	Total Population, *n* = 44
**Gender (*n*, %)**	
Male	19 (43.2)
Female	25 (56.8%)
**Age (mean ± SD in years)**	52.4 ± 11.7
**BMI (*n*, %)**	
≥30	12 (27.3%)
<30	32 (72.7%)
**Calcifications (*n*, %)**	
Complete	7 (15.9%)
Partial	31 (70.5%)
None	6 (13.6%)
**Intraoperative complications**	
Dural tear	2 (4.5%)
Loss of evoked potentials	2 (4.5%)
Massive blood loss (≥1 L)	0
Pleuro-pulmonary dysfunction	0
Pleural tear	0
**Postoperative complications**	
Complete paraplegia	0
Sensory neurological deficit	2 (4.5%)
Surgical site infection	0
CSF fistula	0
**Operative time (mean ± SD in hours)**	4.3 ± 1.1
**Blood loss (mean ± SD in mL)**	107 ± 72
**LOS (mean ± SD in days)**	6.6 ± 7.1
**Follow-up (mean ± SD in months)**	14.2 ± 3.7

**Table 2:** Levels involved.

Thoracic Level	Thoracic Vertebrae Involved	Number of Patients
Upper thoracic spine	T1–T4	4 (9.1%)
Middle thoracic spine	T5–T9	19 (43.2%)
Lower thoracic spine	T9–T12	21 (47.7%)

**Table 3:** Preoperative and postoperative outcomes according to the ASIA impairment scale.

ASIA Impairment Scale	Preoperative *n* (%)	Postoperative (Last FU) *n* (%)
**A**	3 (6.8%)	0 (0%)
**B**	2 (4.5%)	3 (6.8%)
**C**	5 (11.4%)	1 (2.3%)
**D**	12 (27.3%)	7 (15.9%)
**E**	22 (50%)	33 (75%)

## Data Availability

The raw data supporting the conclusions of this article will be made available by the authors on request.

## Abbreviations

TDH	Thoracic disc herniation
DH	Disc herniation
MEPs	Motor evoked potentials
IONM	Intraoperative neurological monitoring
PLL	Posterior longitudinal ligament
LF	Ligamentum flavum
MRA	Magnetic resonance angiography
SSEPs	Somatosensory evoked potentials
